# Spherical Sampler Probes Enhance the Robustness of Ambient Ionization Mass Spectrometry for Rapid Drugs Screening

**DOI:** 10.3390/molecules27030945

**Published:** 2022-01-30

**Authors:** Mariya A. Shamraeva, Denis S. Bormotov, Ekaterina V. Shamarina, Konstantin V. Bocharov, Olga V. Peregudova, Stanislav I. Pekov, Eugene N. Nikolaev, Igor A. Popov

**Affiliations:** 1 Moscow Institute of Physics and Technology, 141701 Dolgoprudny, Russia; shamraeva-masha@mail.ru (M.A.S.); bormotov.ds.ms@gmail.com (D.S.B.); shamarina.ev@phystech.edu (E.V.S.); olga.peregudova@phystech.edu (O.V.P.); 2 V. L. Talrose Institute for Energy Problems of Chemical Physics, N. N. Semenov Federal Center of Chemical Physics, Russian Academy of Sciences, 119334 Moscow, Russia; bo4arow.konstantin@yandex.ru; 3 Skolkovo Institute of Science and Technology, 121205 Moscow, Russia; stanislav.pekov@forwe.ru (S.I.P.); e.nikolaev@skoltech.ru (E.N.N.)

**Keywords:** mass spectrometry, ambient ionization, drug monitoring, emergency room, touch spray ionization, multiple compounds detection

## Abstract

Ambient ionization mass spectrometry has become one of the most promising approaches for rapid and high-throughput screening of small molecules in complex biological matrices for emergency medicine, forensics, and food and agriculture applications. The simple procedures for sample collection and ionization without additional pretreatment are vital in these fields. Many efforts have been devoted to modifying various ambient ionization techniques to simplify the procedures and improve the robustness and sensitivity of the methods. Here, we demonstrate the implementation of rigid spherical sampler probes to improve the robustness of touch spray ionization mass spectrometry. The sphericity of the probes increases the stability of the cone-jet mode of electrospray, reduces the requirements for fine positioning of a sampler in the ion source, and decreases the possibility of corona discharge occurrence. The utilization of spherical sampler probes allows fast, non-invasive sampling, followed by rapid analysis for various drugs of different chemical classes in complex biological matrices, such as the whole blood or sebum collected from the skin surface. The linearity of the analytical signal response from drug concentration confirms the possibility of creating a simple semiquantitative method for small molecules monitoring using spherical sampler probes.

## 1. Introduction

Ambient ionization mass spectrometry has recently become one of the most promising analytical instruments in medical practice [1]. Biomarker screening, intra-surgical monitoring, toxicology, and emergency medicine can benefit significantly from actively developing rapid point-of-care mass spectrometry techniques [1,2,3]. Although conventional gas or liquid chromatography, coupled with mass spectrometry, provides excellent sensitivity, accuracy, and selectivity, the time-consuming steps for sample preparation and separation lengthen an analysis time to over an hour, which is undesirably slow [4], encouraging the development of new high-throughput chromatographic techniques [5], which yet require time for sample preparation. On the other hand, ambient ionization mass spectrometry allows analysis of biological samples in minutes as the aforementioned steps are eliminated from the analysis pipeline [6,7]. The simple procedures for sample collection and further ionization without additional pretreatment make ambient ionization mass spectrometry the method of choice for toxins or overdose screening in an emergency room or resuscitation where rapid analysis is vital [8,9].

The branch of ambient ionization methods has been proposed to analyze various biomolecules in complex matrices, such as saliva, blood, or even biopsy samples without elaborate pretreatment [3,7,10], including desorption electrospray ionization (DESI) [11,12], direct analysis in real time (DART) [13], low-temperature plasma (LTP) [14], desorption atmospheric pressure chemical ionization (DAPCI) [15], a variety of touch spray methods [16,17], and one of the most prominent ambient ionization mass spectrometry methods, paper spray (PS) [18,19,20,21]. Multiple studies have been devoted to investigating the capabilities of PS in performing targeted analysis of substances, such as illicit drugs, therapeutic drugs, metabolites, lipids, and proteins [18,19,21]. PS utilizes chromatographic paper cut into a specific shape, typically a triangle, loaded with dried biological samples and analyzed in a manner similar to electrospray ionization (ESI) by applying a solvent and a high voltage [19]. Thus, the chromatographic paper simultaneously serves as a sampler and ionization source, simplifying the analysis procedure. PS has been implemented in a wide range of applications, including clinical diagnostics [18], forensics [22], toxicology [23], illicit drugs detection [22], population screenings for diseases (e.g., neonatal screening) [24], doping control [21], monitoring of pesticides in food and water [25], and many others [24]. All in all, PS offers many advantages, such as an absence of sample preparation, high throughput, and speed of analysis. Many efforts have been devoted to modifying the PS technique, improving its characteristics, particularly its sensitivity and stability, which is especially important in negative ion mode due to Taylor cone instability and corona discharge occurrence at relatively low voltages [26].

The stability of the ionization process and, thus, the sensitivity of paper spray mass spectrometry is dependent on many conditions, such as the type and shape of paper material, that can affect both the processes of adsorption-desorption and the efficiency of analyte elution [27]. Therefore, many attempts have been made to expand the range of porous materials to improve the characteristics of PS and touch spray methods, based on small probes, such as swabs [16,17] or needles [28]. Using more hydrophobic porous substrates can improve the sensitivity of the method and stability of the signal, especially in negative ion mode [29]. Additionally, polymer materials have shown potential for an ambient ionization mass spectrometry analysis of raw biofluid samples with considerable capacity for yielding quantitative results [27]. Using polypropylene fibers [30], bamboo leaves [31], textile fabrics [32], and others, impressive results have already been provided when studying biological samples [33,34].

**Figure 1:**
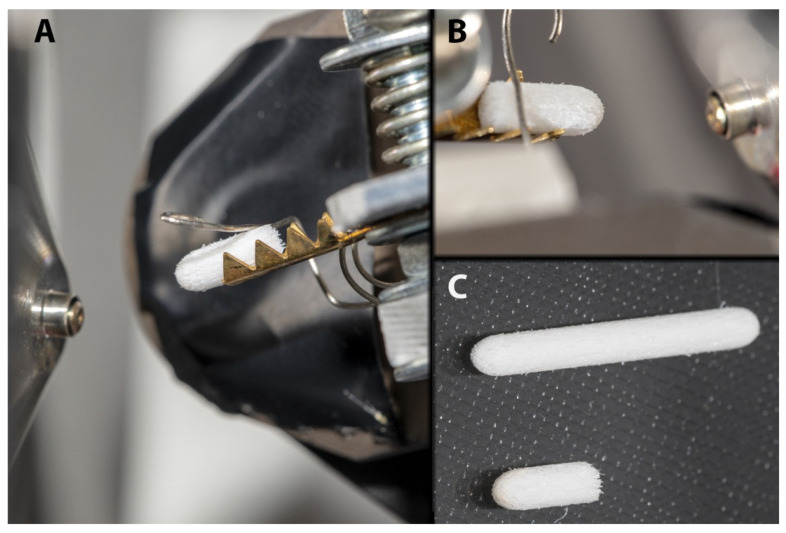
Experimental setup of sampler probe as an ion source. A sampler probe with 10.0 mm length was placed into a brass holder and fixed with a stainless-steel clamp (**A**,**B**). 10.0 mm long sampler probe ((**C**), bottom) was made by cutting commercially available felt-pen tip ((**C**), top).

In the present study, we report a simple and rapid method of determination and quantification of therapeutic drugs through ambient ionization mass spectrometry using a spherical polymer sampler probe (Figure 1), which simplifies sample collection and improves the reliability of the ionization. The characterization of the electrospray ionization process on the spherical sampler demonstrates the high stability of the Taylor cone, which makes the proposed method applicable to the direct analysis of diverse compounds in complex biological matrices with a wide range of applied voltages in positive and negative ion modes.

## 2. Results and Discussion

### 2.1. Characterization of SSP-Emitters for Electrospray Ionization

The current-voltage curve (CVC) can be used to evaluate the robustness of an electrospray-based technique. Ideally, the CVC will exhibit a plateau, as this means that the total ion current remains nearly constant in a wide range of applied voltages. Therefore, the Taylor cone remains stable in a wide range of local electric field strength on the tip, which provides sustainable electrospray ionization [35]. It will, therefore, remain stable if slight changes of the electric field occur due to variations of the distance between the tip and MS inlet or the solvent flow or conductivity. As a result, the conditions for spectra reproducibility become less strict, making the ambient ionization method easier to implement and use. In the case of current strongly depending on voltage, small changes in any of the aforementioned parameters, as well as uncontrollable changes in airflow, could alter the electrospray regime, reducing spectra reproducibility and, in some situations, causing corona discharge.

**Figure 2:**
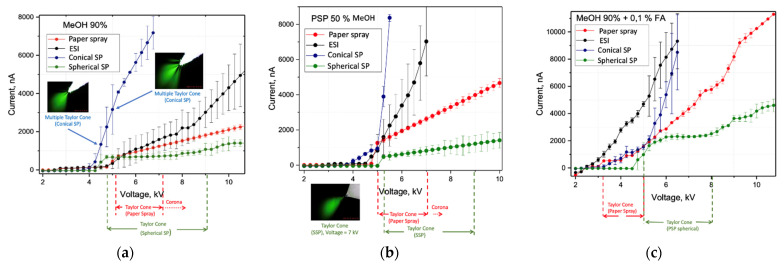
Electrospray current as a function of applied voltage in the range 2–10.5 kV for different solvents (positive ion mode): (**a**) 9:1 (*v*/*v*) MeOH: H_2_O; (**b**) 1:1 (*v*/*v*) MeOH: H_2_O; (**c**) 9:1 (*v*/*v*) MeOH: H_2_O + 0.1% FA (*v*/*v*). The distance between the tip of the ion source and the counter electrode is 10.0 mm, angle 30°.

**Figure 3:**
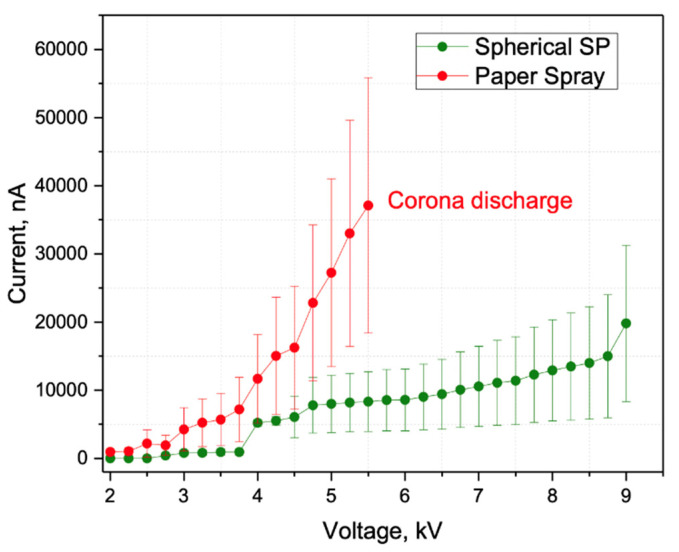
Electrospray current as a function of applied voltage in the range 2–9 kV (negative ion mode). Solvent—1:1 (*v*/*v*) MeOH:i-PrOH. The distance between the tip of the ion source and the counter electrode is 10.0 mm, angle 30°.

For most solvents, the electrospray current initiated at a voltage of about 5.0 kV and increased and leveled off at about 200 nA. Further, a plateau behavior occurred in a wide range between 6.5 kV and 8 kV—the electrospray current was nearly independent of the applied voltage, which is a well-known characteristic of the cone-jet mode of electrospray (Figure 2 and Figure 3, and Figures S2 and S3 in Supplementary Materials). The plateau continued until a voltage of 8 kV was applied, where the current increases rapidly, resulting in the initiation of corona discharge. The experiments demonstrated that the sampler probe of spherical shape had a wider range of work voltage than the porous sampler probe of conical shape or the PS, which means a more robust ionization process compared to the PS where the increase in current has no subsequent plateau range.

Because of the greater volume-to-surface ratio, the sampler probe of spherical shape as an ion source is much less sensitive to many factors, including the solvent flow rate, because evaporation processes are less prevalent, which makes a wider range of flow rates suitable before running into significant instability problems, especially compared to in PS, where flat paper is used [36]. Thus, the sampler probe enables the acquisition of stable signals over extended periods. It should be noted that SSP demonstrates higher RSD at relatively high voltages than PS. Such behavior may somewhat reduce the ionization stability, but the observed current remains substantially lower. This drastically decreases the possibility of corona discharge occurrence, which could be a reason for paper smoldering in the case of PS. After that, the plateau region on CVC for SSP remains wider than for PS for all tested solvent mixtures, so a broader deviation in the geometry and positioning of the sampler in the ion source is acceptable.

**Figure 4:**
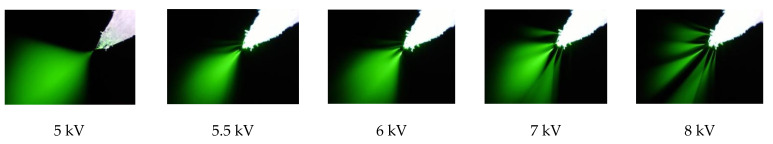
The photos of sharply angled marker tips (conical) in the range of potential 5–8 kV. Multiple Taylor cones formation from sharply angled marker tips.

**Figure 5:**
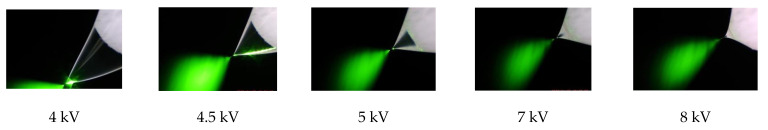
The photos of round marker tips in the range of potential 4–8 kV. Single Taylor cone formation.

Comparing pointed and round samplers revealed that the latter is better suited for consistently obtaining single cone-jet electrospray. Pointed samplers, as well as PS samplers [34], have a higher local electric field on the tip in the absence of solvent flow and tend to have multiple loose fibers where the local electric field may be even higher. This results in a less predictable MultiJet regime (see Figure 4) or corona discharge at much lower spray voltages compared to similar experiments with round tip samplers (see Figure 5). This result is not restricted to proposed polymer fiber samplers but may be eventually expanded for various touch spray methods.

It is worth mentioning that a corona discharge also occurred at high voltages (>8 kV) on round marker tip samplers. The discharge may also be used to initiate atmosphere pressure chemical ionization to facilitate the detection of non-polar compounds [37]. Such analytes are practically impossible to detect utilizing PS, where initiation of corona discharge at high voltages (more than 6 kV) results in paper smoldering [26].

### 2.2. The Analysis of Drugs Using Sampler Probes of Spherical Shape

The direct analysis using sampler probes allows combining sample collection procedures with the following ionization of the samples. The model drugs with different physical and chemical properties were analyzed and characterized using tandem mass spectrometry followed by quantitative analytical performance measurements. The acetaminophen, papaverine, and diclofenac could be ionized in positive ion mode because of nucleophilic properties, preferring to attack *N*-heterocyclic groups. On top of that, papaverine is an opium alkaloid, detection of which shows the potential of the method for identification of other opium alkaloids, such as morphine, codeine, thebaine, and noscapine. Ibuprofen and diclofenac could be detected in negative ion mode because they have acidic properties, involving a loss of H^+^ from a carboxylic group. Moreover, the development of ambient mass spectrometry methods working in negative ion mode with good sensitivity is extremely pertinent.

**Figure 6:**
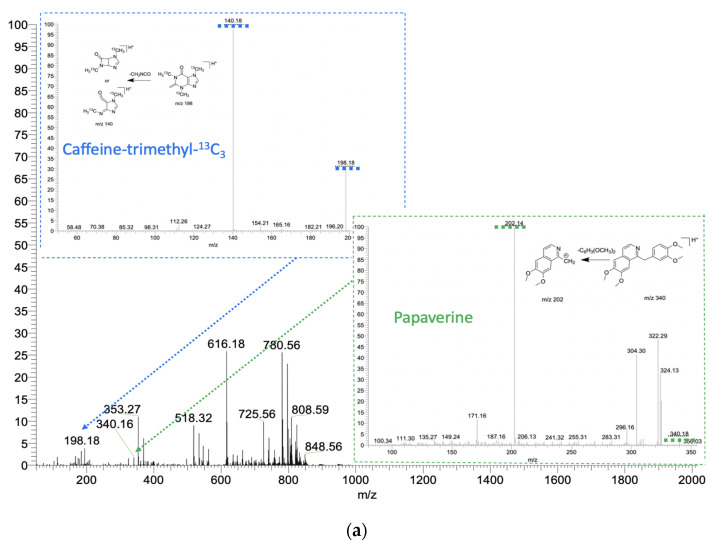

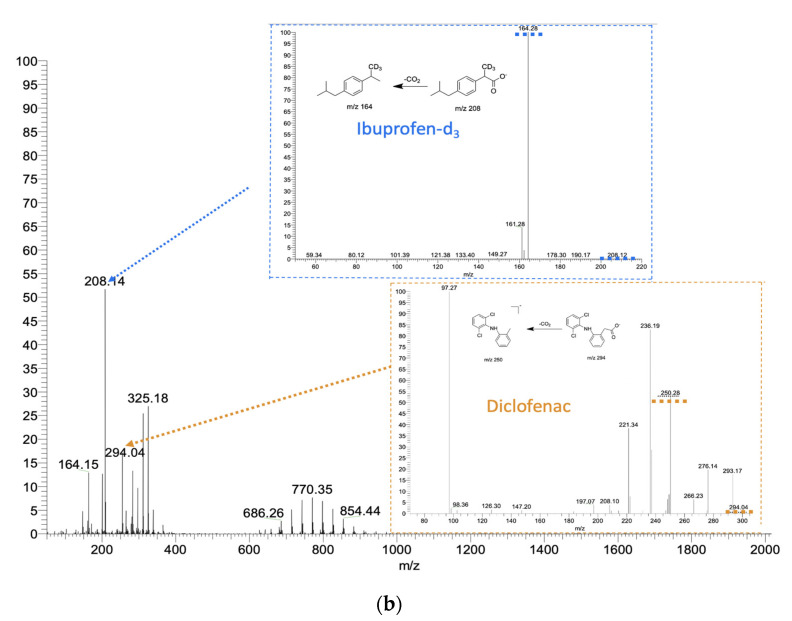
(**a**) Mass spectra of drugs (papaverine 2500 ng/mL and caffeine-trimethyl-^13^C_3_ a 100 µg/mL as an internal standard) in blood in positive ion mode. Spray solvent is 9:1 (*v*/*v*) methanol:H_2_O with 0.1% (*v*/*v*) formic acid. Spray voltage is 5 kV. (**b**) Mass spectra of drugs (diclofenac 2000 ng/mL and ibuprofen-*d*_3_ 100 µg/mL as an internal standard) in blood in negative ion mode. Spray solvent is 1:1 (*v*/*v*) methanol: 2-propanol. Spray voltage is 4.5 kV.

**Figure 7:**
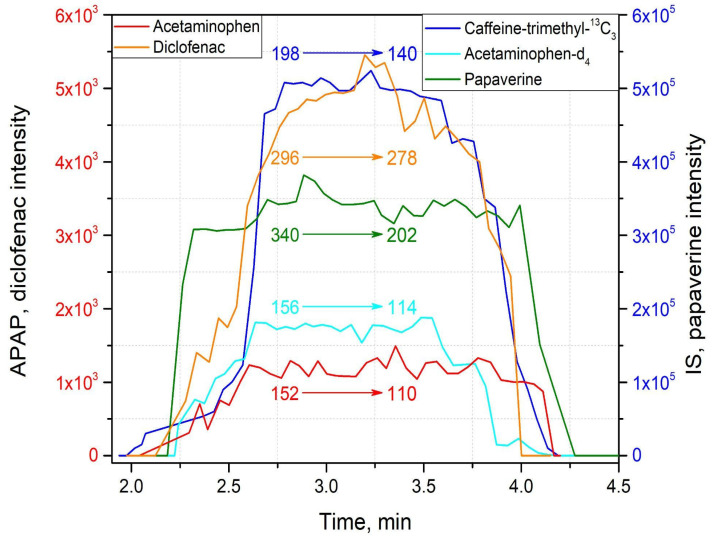
Extracted ion chronograms (EICs) obtained for acetaminophen (2500 ng/mL), diclofenac (2500 ng/mL), papaverine (2500 ng/mL) and internal standards caffeine-trimethyl-^13^C_3_ (100 µg/mL) and acetaminophen-*d*_4_ (100 µg/mL) in positive ion mode for determination of LOD.

**Figure 8:**
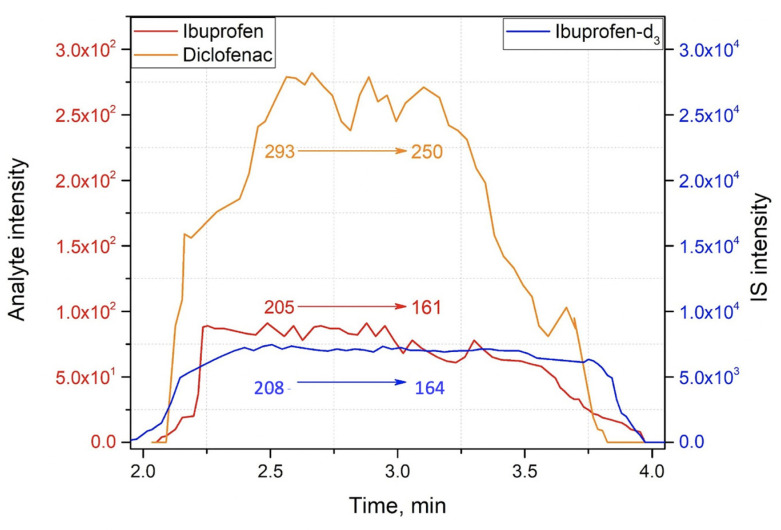
EICs obtained for ibuprofen (2000 ng/mL), diclofenac (2000 ng/mL) and internal standard ibuprofen-*d*_3_ (100 µg/mL) in negative ion mode for determination of LOD.

The method capabilities were evaluated using model drugs standards (4 μL of the standards mixture) which were loaded onto an SSP with a spherical shape surface and left to dry completely at room temperature. Then all the SSP were spiked with a 100 μg/mL solution of internal standards—caffeine-trimethyl-^13^C_3_, acetaminophen-*d*_4_ for positive mode and ibuprofen-*d*_3_ as an internal standard for negative mode. The second similar set of samples was prepared using blood as the matrix. Ambient ionization methods are intended to be implemented to untreated samples, and blood represents one of the most complicated matrices, containing a wide variety of molecules belonging to different chemical classes, which, alongside with physical properties of blood, could significantly affect the ionization process. The blood samples were prepared by dripping 4 μL of the blood of healthy volunteers. Without letting them dry, 4 μL of the model drug standards of known concentration were added onto an SSP and dried completely under room conditions. Afterward, 4 μL of internal standards—100 μg/mL of caffeine-trimethyl-^13^C_3_, 100 μg/mL of acetaminophen-*d*_4_ for positive mode, and 100 μg/mL of ibuprofen-*d*_3_ for the negative mode—were loaded onto the SSP with blood samples and dried within 5 min. So, both spiked matrices, pure solvent and blood, contained model drug standards in the concentration range from 100 ng/mL to 2750 ng/mL, and identical amounts of internal standards were deposited on the SSP. Then 30 μL of MeOH/H2O (9:1, *v*/*v*), doped with 0.1% (*v*/*v*) formic acid or 90 μL of 1:1 (*v*/*v*) MeOH:i-PrOH, were manually deposited by pipette to perform the acquisition in positive or negative mode, respectively. Matrix effects for APAP and diclofenac were calculated for two concentrations, 250 ng/mL and 2000 ng/mL. Tandem mass spectrometry was used to confirm the structures of analyzed therapeutic drugs, and the analytical performance of the method, exploiting the sampler probe with spherical shape, was carried out in MS/MS mode, monitoring the following transitions in positive mode: *m*/*z* 198⮞140 for caffeine-trimethyl-^13^C_3_; *m*/*z* 156⮞114 for acetaminophen-*d*_4_ (internal standards); *m*/z 296⮞278 for diclofenac; *m*/*z* 340⮞202 for papaverine; *m*/*z* 152⮞110 for acetaminophen, and, in negative mode: *m*/*z* 208⮞164 for ibuprofen-*d*_3_ (internal standard); *m*/*z* 205⮞161 for ibuprofen; and *m*/*z* 294⮞250 for diclofenac (Figure 6, Figures S4 and S5, and Table S1 in Supplementary Materials). Under these conditions, distinct peaks persisting for most of the duration of the experiment were observed in the extracted ion chronograms (EIC) of fragment ions. The representative EIC, acquired during 120 s, which was required for complete elution of the added solvent from the SSP, for positive and negative modes are shown in Figure 7 and Figure 8, respectively.

**Figure 9:**
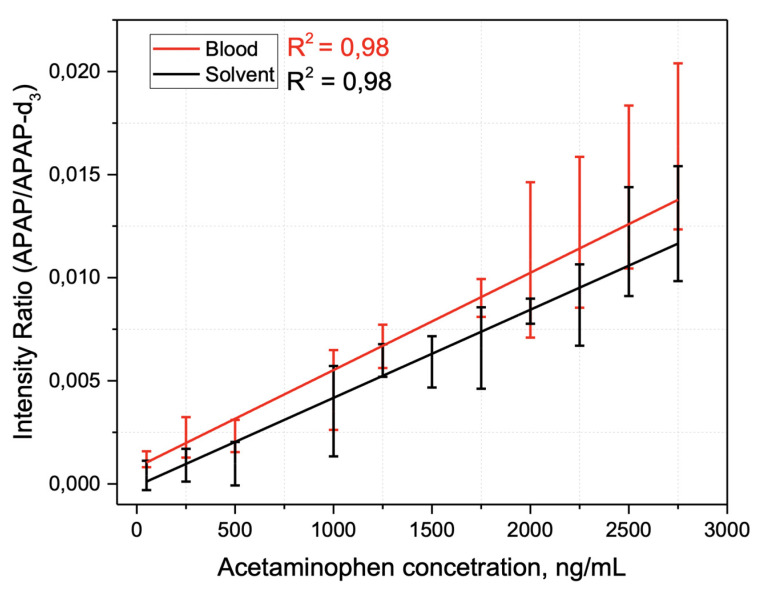
Calibration curves in positive mode (*p*-value < 0.05): black: acetaminophen with APAP-*d*_3_ as an internal standard. Matrix: 9:1 (*v*:*v*) MeOH:H_2_O; R^2^ = 0.98. red: papaverine with caffeine-trimethyl-^13^C_3_ as an internal standard. Matrix: dried blood; R^2^ = 0.98.

The calibration curves for quantitation were constructed by averaging (*n* = 3) the experimental areas under the curves of the transitions of analyzed therapeutic drugs to the transitions of IS plotted against known concentrations of analyzed drugs with a minimum of 6 points over a linear dynamic range of concentration. The IS was used to compensate for deviations in instrumental responses caused by different sample deposition. The pioneer studies of pharmaceuticals in biological matrices utilizing paper spray have shown the magnitude of IS application [38,39]. The calibration curves obtained for acetaminophen in two matrices—solvent and blood—with APAP-*d*_3_ as the internal standard are shown in Figure 9. The R^2^ value was 0.98 in solvent and 0.98 in blood over the concentration range of 50–2750 ng/mL, which is tolerable for a rough concentration estimation and confirms the linear response of an analytical signal from concentration. This achieved linear range is comparable to the previously described method of solid-phase microextraction coupled with thermal desorption electrospray ionization mass spectrometry [40] and shows the suitability of the described method with a porous sampler probe for direct analysis of raw biological samples.

The direct analysis of analytes using a sampler probe without time-consuming sample preparation and chromatographic separation provided the following LOD values (calculated as a fragment ion signal more than three times greater than the standard deviation over the noise plus the mean value of the blank, in MS/MS mode): 10 ng/mL and 10 pg/mL for acetaminophen and papaverine in positive mode and solvent as a matrix, 2 µg/mL and 250 pg/mL for acetaminophen and papaverine, respectively, in positive mode and blood as a matrix (Figure 9 and Figure S6 in Supplementary Materials); 5 µg/mL and 5 ng/mL for diclofenac and ibuprofen in solvent, respectively, and LOQ values for acetaminophen and papaverine in positive mode were 33 ng/mL and 33 pg/mL in solvents as matrix and 7 µg/mL and 833 pg/mL in blood. The LOQ values in negative ion mode were 17 µg/mL and 17 ng/mL for diclofenac and ibuprofen. A linear dynamic concentration range varied in the range of 2 to 3 orders of magnitude (Figure S7 in Supplementary Materials). These results are in good agreement with the therapeutic concentrations range, demonstrating the method’s suitability for therapeutic monitoring of drugs in biological matrices [41] and comparable with conventional methods, such as LC-MS/MS [42,43,44], or methods with preliminary extraction [40]. Matrix effects for APAP were calculated for two concentrations, 250 ng/mL (80.5%) and 2000 ng/mL (79.8%), and for diclofenac were 250 ng/mL (101.2%) and 2000 ng/mL (122.9%). Strong negative matrix effects, meaning severe ion suppression, were not found for either APAP or diclofenac.

To evaluate the reproducibility of the method, a ratio of signals of two internal standards was used. In positive mode, a number of measurements were obtained with the same concentrations of the two internal standards: 100 µg/mL for both caffeine-trimethyl-^13^C_3_ and acetaminophen-*d*_4_. Ideally, the ratio between peak areas of the corresponding peaks should remain constant in these experiments. We obtained the ratio values for 114 probes over 5 non-consecutive days. The average ratio of product ion peak areas (acetaminophen-*d*_4_/caffeine-trimethyl-^13^C_3_) was 0.31 ± 0.08. The relative standard deviation (RSD) of 26% is comparable with swab touch electrospray [45] and paper spray [22] methods and indicates that the reproducibility of the method is high enough for semiquantitative analysis. Notably, the inter-day variability, expressed as a standard deviation between averages of measurements obtained in a particular day was much lower than the overall standard deviation; it was 0.02 (or 7% if expressed as RSD), so the factors that changed between days (such as ambient temperature) did not contribute significantly to the method reproducibility. Considering this, the relatively high RSD probably stems from the technical imperfection of the implemented setup. Since the solvent is added on the sampler manually by the operator, minor changes in the geometry of the Taylor cone and the jet of droplets are inevitable in this design.

### 2.3. Method Application

**Figure 10:**
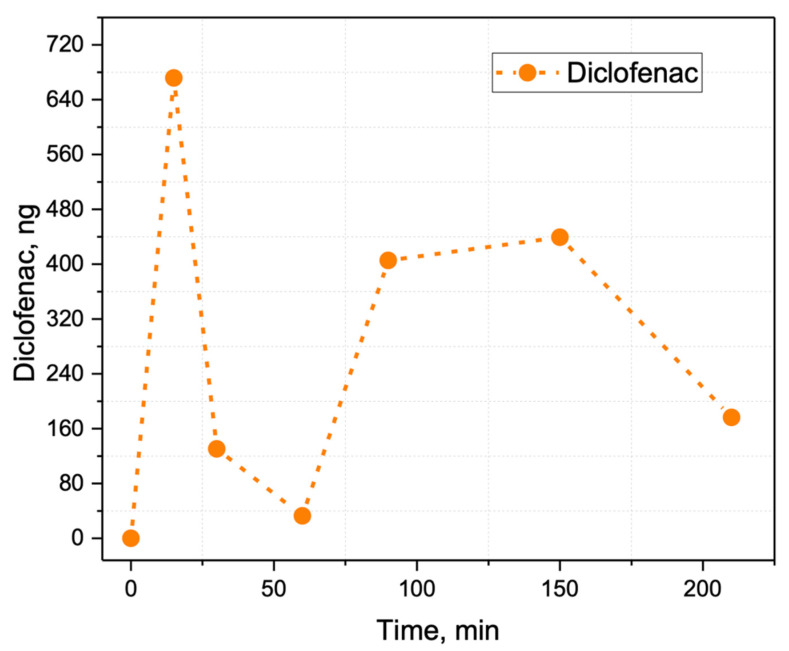
Relative change in diclofenac content in human sebum after applying a diclofenac salve. At 15 min, the diclofenac was detected as it was released from the blood while transporting from the application area. After 60 min, the diclofenac accumulated in synovial fluid releases through the skin.

To simulate non-invasive drug detection from the skin surface, diclofenac was detected on the probes brought into contact with the skin of a volunteer. More specifically, 3.5 g of 5% diclofenac salve was applied to the volunteer’s inner forearm (Figure S8a in Supplementary Materials). The samples were taken from the elbow with precautions taken to avoid accidentally touching the inner forearm. A sample was taken by scraping the elbow area 10 times in a straight line about 3 cm in length. It is worth mentioning that, due to mechanical rigidity, the sampler probes proposed in this study were easy to manipulate and allowed for controlled sampling from the skin surface, while paper samplers would crumple uncontrollably if a similar procedure was attempted. This procedure was repeated with clean probes at 0, 15, 30, 60, 90, 150, and 210 min after the salve was applied, and then the ratio of diclofenac and ibuprofen-*d*_3_ product ions peak areas was measured for each probe. The resulting ratios are shown on Figure 10 and indicate that the diclofenac quantities on the probes were well above detection and quantitation limits. Notably, the time dependence had two maximums; the first maximum (680 ng of diclofenac on the SSP) may be attributed to the analyte being carried via blood directly from the application area, and the second (440 ng on the SSP) may be attributed to diclofenac accumulation in the synovial fluid in the elbow [46]. Despite positive matrix effects for diclofenac, which could also occur in the case of sebum matrix (Figure S8 in Supplementary Materials), the miniscule and non-identical weight of the collected samples, which depends on the pressure and actual track of the sampler probe during the scratching, the relative change of the diclofenac quantity in sebum could be estimated. The quantity of the analyte molecules on the probe was roughly proportional to their surface concentration on the skin between the experiments since the sampling was performed according to one scratching scheme.

## 3. Materials and Methods

### 3.1. Materials

Methanol (MeOH, LiChrosolv, LC gradient grade) and 2-propanol (i-PrOH, LiChrosolv, LC gradient grade) were purchased from Merck KGaA (Darmstadt, Germany); acetaminophen (APAP, *N*-acetyl-4-aminophenol, paracetamol, 4-acetamidophenol) and ibuprofen (α-methyl-4-(isobutyl)phenylacetic acid) were purchased from Merck KGaA (Darmstadt, Germany). Caffeine-trimethyl-^13^C_3_, *N*-(4-hydroxyphenyl-2,3,5,6-*d*_4_)-acetamide (4-acetamidophenol-*d*_4_, acetaminophen-d_4,_ APAP-*d*_4_) and ibuprofen-*d*_3_ (α-methyl-*d*_3_-4-(isobutyl)phenylacetic acid) were purchased from Toronto Research Chemicals (Toronto, ON, Canada). Formic acid (FA, MS grade) was obtained from Merck KGaA (Darmstadt, Germany). The therapeutic drug, acetaminophen (APAP, *N*-acetyl-p-aminophenol, or paracetamol) was purchased from the pharmaceutical company Renewal (Novosibirsk, Russia). Papaverine (6,7-dimethoxy-1-veratrylisoquinoline hydrochloride), in the form of the hydrochloride, was purchased from the pharmaceutical company “Pharmstandard” (Dolgoprudny, Russia), and diclofenac from the pharmaceutical company “Synthesis” (Kurgan, Russia) (2-[2-(2,6-dichloroanilino)phenyl]acetic acid) and ibuprofen was purchased from the pharmaceutical company “Borisov Plant of Medical Preparations” (Belarus, Borisov) and used without further treatment. The spherical sampler probe (SSP) is, in essence, rigid rods of a compressed longitudinally elongated polymer fiber. In this study, we used low-density polypropylene fiber rods with spherical and conical shapes, which are commercially available as Molotow™ felt-pen tips, and chromatography paper (Whatman™ cat. no. 3001-861). The working solution consisting of 9:1 (*v*/*v*) MeOH:H_2_O methanol:H_2_O with 0.1% (*v*/*v*) formic acid and the working solution consisting of 1:1 (*v*/*v*) MeOH: i-PrOH was found to be altogether suitable for the analysis and for elution from sampler probes in positive ion mode and in negative ion mode, respectively. The solutions of acetaminophen-*d*_4_, caffeine-trimethyl-^13^C_3_ and ibuprofen-*d*_3_ 100 µg/mL were prepared in methanol and stored at −20 °C for further use as internal standards (IS). The stock solutions of acetaminophen (1 mg/mL), papaverine (1 mg/mL), diclofenac (1 mg/mL) and ibuprofen (1 mg/mL) were prepared in methanol and stored at −20 °C. The venous blood samples were collected from healthy volunteers using EDTA-2K vacuum blood collection tubes (Improvacuter, Guangzhou Improve Medical Instruments Co., Ltd., Guangzhou, China) and incubated at room temperature for 15 min before further manipulations. The calibration solutions of acetaminophen, papaverine and diclofenac were prepared by serial dilution of the stock solution with methanol. Calibration standards of acetaminophen, papaverine, diclofenac and ibuprofen were prepared at concentrations of 100 to 2750 ng/mL.

### 3.2. Determination of the Current–Voltage Curve

To obtain a current-voltage curve (CVC), a setup was built to imitate electrospray conditions (Figure S1 in Supplementary Materials). The solvent flow was controlled using a Cole-Parmer^®^ syringe pump (Masterflex Single-Syringe Infusion Pump; 230 VAC, Item # GY-74900-05, Masterflex, Radnor, PA, USA), with the syringe needle piercing the back of the marker tip, which was used as an electrode set up at a fixed distance from the counter electrode. The DC high voltage (HV) was provided by a Mantigora HT-60-30-P high voltage source (Mantigora, Novosibirsk, Russia) applied to the marker tip for positive mode measurements or to the counter-electrode for negative mode measurements. The other electrode was connected to the ground through 100 MΩ resistance. The electrospray current was measured indirectly as a voltage between the ground and the electrode that was not the one with HV applied, divided by the 100 MΩ.

### 3.3. Mass Spectrometry

All experiments were performed using the LTQ XL Orbitrap ETD hybrid mass spectrometer (ThermoFisher, San Jose, CA, USA) in full scan mode from *m/z* 50–1000 and MS^2^ mode. In both modes, the MS inlet temperature was set to 220 °C. The marker tip was placed into a metallic holder attached to a three-dimensional positioning system, which ensured that the geometric parameters of the source were reproducible within approximately 0.2 mm. Two cameras attached to the same system were used for control of the marker position and spectra stability. A sampler probe of 10.0 mm length was used as a substrate and carrier for electrospray ionization. The sample was first preloaded on the surface substrate and dried. The raw blood was firstly preloaded by dripping 4 µL of the whole blood samples onto the porous sampler probe mount, with subsequent air drying for 2 h before the acquisition of MS spectra.

To implement the electrospray ionization, the solvent was deposited manually using a pipette (30 µL and 90 µL of working solutions in positive and negative mode, respectively), and a high voltage (DC) was applied to the marker tip through the metallic holder. With the increase in voltage, as well as wicking, the solvent containing analytes transfer to the marker tip, where due to the strong electric field electrospray ionization takes place. The MS analysis was carried out in positive ion mode with a spray voltage of 5.0 kV, and in negative ion mode with a spray voltage of 4.5 kV. The distance between the MS inlet and the probe tip (at the closest point) was 7.5 mm and 13 mm for positive and negative ion mode measurements, respectively (Figure 1). Full MS spectra were obtained using an Orbitrap mass analyzer, with resolution 30,000 FWHM at *m*/*z* = 400. MS^2^ spectra were obtained using the LTQ mass analyzer at a “normal” scan rate and collision-induced dissociation (CID) isolation window width was 2.0 Da with normalized activation energy 35.0 eV.

### 3.4. Characterization

Linearity of instrument standard response was determined for all analytes over the range of concentrations from 100 to 2750 ng/mL. The limit of detection (LOD) and the limit of quantitation (LOQ) were determined as the concentration that produced a fragment ion signal more than three and ten times, respectively, greater than the standard deviation over the noise plus the mean value of the blank in the MS/MS mode using CID. Calibration curves were fitted by plotting peak area ratios (monitored transitions of analytes/IS) against nominal concentrations of the calibration standards. The quantitative measurements were performed three times by spiking (4 µL) of the IS solution to the samples and with the appropriate amount of working solutions 30 µL and 90 µL in positive and negative mode, respectively. The calibration curves for all analytes were fitted using weighted least-squares linear regression with a weighting factor of 1/x^2^. The R^2^ values were used to roughly estimate linearity. Matrix effects for APAP and diclofenac were calculated as the ratio of the average normalized area of three spiked blood samples and the average normalized area of solutions prepared in used solvents.

## 4. Conclusions

The rigid spherical sampler probes simplify the manipulations during sample collection and ionization, providing a convenient analytical strategy for rapid screening of small molecules in complex biological matrices, such as sebum or blood. The spherical form of the sampler probes led to improved stability of the cone-jet mode of electrospray, which decreased the requirements for fine voltage adjustment and the positioning of the samplers in the ion source. Additionally, spherical sampler probes demonstrated robust ionization in negative mode compared to other ambient ionization methods, such as paper spray, significantly expanding the variety of analyzed compounds. This result is not restricted to proposed polymer fiber samplers but may eventually be expanded for various touch spray methods.

The analysis of a set of therapeutic drugs belonging to different chemical classes demonstrates the high sensitivity of the proposed method. The determined detection and quantitation limits were below the therapeutic range of the analyzed drugs and even comparable with the sensitivity of conventional HPLC-MS/MS methods. A linear response in the range of two to three orders of magnitude for every compound suggests that porous sampler probes are also helpful for semiquantitative touch spray analysis. These features make the proposed technique particularly useful for rapid analysis of toxins or overdose screening in an emergency room or resuscitation.

## Data Availability

The data related to this study are available from the corresponding author upon reasonable request.

## Supplementary Materials

The following supporting information can be downloaded in a combined file: Supporting Figures S1–S8 (as referenced in main text) and a Supporting Table S1: Analyzed chemical structures and monitored product ions.

## References

[1] Feider C.L., Krieger A., DeHoog R.J., Eberlin L.S. (2019). Ambient Ionization Mass Spectrometry: Recent Developments and Applications. Anal. Chem..

[2] Ferreira C.R., Yannell K.E., Jarmusch A.K., Pirro V., Ouyang Z., Cooks R.G. (2016). Ambient Ionization Mass Spectrometry for Point-of-Care Diagnostics and Other Clinical Measurements. Clin. Chem..

[3] Pekov S.I., Bormotov D.S., Nikitin P.V., Sorokin A.A., Shurkhay V.A., Eliferov V.A., Zavorotnyuk D.S., Potapov A.A., Nikolaev E.N., Popov I.A. (2021). Rapid estimation of tumor cell percentage in brain tissue biopsy samples using inline cartridge extraction mass spectrometry. Anal. Bioanal. Chem..

[4] Lee Y.W. (2013). Simultaneous Screening of 177 Drugs of Abuse in Urine Using Ultra-performance Liquid Chromatography with Tandem Mass Spectrometry in Drug-intoxicated Patients. Clin. Psychopharmacol. Neurosci..

[5] Fialkov A.B., Lehotay S.J., Amirav A. (2020). Less than one minute low-pressure gas chromatography—Mass spectrometry. J. Chromatogr. A.

[6] Ma X., Ouyang Z. (2016). Ambient ionization and miniature mass spectrometry system for chemical and biological analysis. Trends Analyt. Chem..

[7] Lawton Z.E., Traub A., Fatigante W.L., Mancias J., O’Leary A.E., Hall S.E., Wieland J.R., Oberacher H., Gizzi M.C., Mulligan C.C. (2017). Analytical Validation of a Portable Mass Spectrometer Featuring Interchangeable, Ambient Ionization Sources for High Throughput Forensic Evidence Screening. J. Am. Soc. Mass Spectrom..

[8] Lee C.W., Su H., Cai Y.D., Wu M.T., Wu D.C., Shiea J. (2017). Rapid Identification of Psychoactive Drugs in Drained Gastric Lavage Fluid and Whole Blood Specimens of Drug Overdose Patients Using Ambient Mass Spectrometry. Mass Spectrom..

[9] Lee C.W., Chao Y.Y., Shiea J., Shen J.H., Lee H.H., Chen B.H. (2018). Ambient mass spectrometry for rapid diagnosis of psychoactive drugs overdose in an unstable patient. Am. J. Emerg. Med..

[10] Maher S., Jjunju F., Taylor S. (2015). Colloquium: 100 years of mass spectrometry: Perspectives and future trends. Rev. Mod. Phys..

[11] Takáts Z., Wiseman J.M., Gologan B., Cooks R.G. (2004). Mass spectrometry sampling under ambient conditions with desorption electrospray ionization. Science.

[12] Eberlin L.S. (2014). DESI-MS imaging of lipids and metabolites from biological samples. Methods Mol. Biol..

[13] Smoluch M., Mielczarek P., Silberring J. (2016). Plasma-based ambient ionization mass spectrometry in bioanalytical sciences. Mass Spectrom. Rev..

[14] Harper J.D., Charipar N.A., Mulligan C.C., Zhang X., Cooks R.G., Ouyang Z. (2008). Low-temperature plasma probe for ambient desorption ionization. Anal. Chem..

[15] Smith B.L., Hughes D.M., Badu-Tawiah A.K., Eccles R., Goodall I., Maher S. (2019). Rapid Scotch Whisky Analysis and Authentication using Desorption Atmospheric Pressure Chemical Ionisation Mass Spectrometry. Sci. Rep..

[16] Morato N.M., Pirro V., Fedick P.W., Cooks R.G. (2019). Quantitative Swab Touch Spray Mass Spectrometry for Oral Fluid Drug Testing. Anal. Chem..

[17] Patrick W.F., Ryan M.B. (2017). Swab touch spray mass spectrometry for rapid analysis of organic gunshot residue from human hand and various surfaces using commercial and fieldable mass spectrometry systems. Forensic Chem..

[18] Lin C.H., Liao W.C., Chen H.K., Kuo T.Y. (2014). Paper spray-MS for bioanalysis. Bioanalysis.

[19] Manicke N.E., Bills B.J., Zhang C. (2016). Analysis of biofluids by paper spray MS: Advances and challenges. Bioanalysis.

[20] Vandergrift G.W., Hessels A.J., Palaty J., Krogh E.T., Gill C.G. (2018). Paper spray mass spectrometry for the direct, semi-quantitative measurement of fentanyl and norfentanyl in complex matrices. Clin. Biochem..

[21] Rossini E.L., Kulyk D.S., Ansu-Gyeabourh E., Sahraeian T., Pezza H.R., Badu-Tawiah A.K. (2020). Direct Analysis of Doping Agents in Raw Urine Using Hydrophobic Paper Spray Mass Spectrometry. Mass Spectrom..

[22] Teunissen S.F., Fedick P.W., Berendsen B.J.A., Nielen M.W.F., Eberlin M.N., Graham Cooks R., van Asten A.C. (2017). Novel Selectivity-Based Forensic Toxicological Validation of a Paper Spray Mass Spectrometry Method for the Quantitative Determination of Eight Amphetamines in Whole Blood. J. Am. Soc. Mass Spectrom..

[23] McKenna J., Jett R., Shanks K., Manicke N.E. (2018). Toxicological Drug Screening using Paper Spray High-Resolution Tandem Mass Spectrometry (HR-MS/MS). J. Anal. Toxicol..

[24] Frey B.S., Damon D.E., Badu-Tawiah A.K. (2020). Emerging trends in paper spray mass spectrometry: Microsampling, storage, direct analysis, and applications. Mass Spectrom. Rev..

[25] Jjunju F.P.M., Damon D.E., Romero-Perez D., Young I.S., Ward R.J., Marshall A., Maher S., Badu-Tawiah A.K. (2020). Analysis of non-conjugated steroids in water using paper spray mass spectrometry. Sci. Rep..

[26] Espy R.D., Muliadi A.R., Ouyang Z., Cooks R.G. (2012). Spray mechanism in paper spray ionization. Int. J. Mass Spectrom..

[27] Yang Q., Wang H., Maas J.D., Chappell W.J., Manicke N.E., Cooks R.G., Ouyang Z. (2012). Paper spray ionization devices for direct, biomedical analysis using mass spectrometry. Int. J. Mass Spectrom..

[28] Kerian K.S., Jarmusch A.K., Cooks R.G. (2014). Touch spray mass spectrometry for in situ analysis of complex samples. Analyst.

[29] Wong M.M., Man S.H., Che C.M., Lau K.C., Ng K.C. (2014). Negative electrospray ionization on porous supporting tips for mass spectrometric analysis: Electrostatic charging effect on detection sensitivity and its application to explosive detection. Analyst.

[30] Aquino A., Mayrink Alves Pereira G., Dossi N., Piccin E., Augusti R. (2021). Reagent-Pencil and Paper Spray Mass Spectrometry: A Convenient Combination for Selective Analyses in Complex Matrixes. J. Am. Soc. Mass Spectrom..

[31] Li X., Tao W., Xun H., Yao X., Wang J., Sun J., Yue Y., Tang F. (2021). Simultaneous Determination of Flavonoids from Bamboo Leaf Extracts Using Liquid Chromatography-Tandem Mass Spectrometry. Rev. Bras. Farmacogn..

[32] Cochran K.H., Barry J.A., Muddiman D.C., Hinks D. (2013). Direct analysis of textile fabrics and dyes using infrared matrix-assisted laser desorption electrospray ionization mass spectrometry. Anal. Chem..

[33] Filho J.F.A., Dos Santos N.A., Borges K.B., Lacerda V., Pelição F.S., Romão W. (2020). Fiber spray ionization mass spectrometry in forensic chemistry: A screening of drugs of abuse and direct determination of cocaine in urine. Rapid Commun. Mass Spectrom..

[34] Levin R.E., Shamraeva M.A., Larina I.M., Bormotov D.S., Pekov S.I., Shivalin A.S., Silkin S.V., Eliferov V.A., Bocharov K.V., Nikolaev E.N. (2021). The Development of a method for direct mass spectrometry analysis of biological samples using porous samplers. Aerospace Environ. Med..

[35] Tepper G., Kessick R. (2009). Nanoelectrospray aerosols from microporous polymer wick sources. Appl. Phys. Lett..

[36] Liu J., Wang H., Manicke N.E., Lin J.M., Cooks R.G., Ouyang Z. (2010). Development, characterization, and application of paper spray ionization. Anal. Chem..

[37] Cheng S.C., Jhang S.S., Huang M.Z., Shiea J. (2015). Simultaneous detection of polar and nonpolar compounds by ambient mass spectrometry with a dual electrospray and atmospheric pressure chemical ionization source. Anal. Chem..

[38] Manicke N.E., Abu-Rabie P., Spooner N., Ouyang Z., Cooks R.G. (2011). Quantitative analysis of therapeutic drugs in dried blood spot samples by paper spray mass spectrometry: An avenue to therapeutic drug monitoring. J. Am. Soc. Mass Spectrom..

[39] Manicke N.E., Yang Q.A., Wang H., Oradu S., Zheng O.Y., Cooks R.G. (2011). Assessment of paper spray ionization for quantitation of pharmaceuticals in blood spots. Int. J. Mass Spectrom..

[40] Shiea J., Bhat S.M., Su H., Kumar V., Lee C.W., Wang C.H. (2019). Rapid quantification of acetaminophen in plasma using solid-phase microextraction coupled with thermal desorption electrospray ionization mass spectrometry. Rapid Commun. Mass Spectrom..

[41] Schulz M., Iwersen-Bergmann S., Andresen H., Schmoldt A. (2012). Therapeutic and toxic blood concentrations of nearly 1000 drugs and other xenobiotics. Crit. Care.

[42] Yang Y.J., Liu X.W., Kong X.J., Qin Z., Li S.H., Jiao Z.H., Li J.Y. (2019). An LC-MS/MS method for the quantification of diclofenac sodium in dairy cow plasma and its application in pharmacokinetics studies. Biomed. Chromatogr..

[43] Alam M.A., Al-Jenoobi F.I., Al-Mohizea A.M. (2015). High-Throughput Ultra-Performance LC–MS-MS Method for Analysis of Diclofenac Sodium in Rabbit Plasma. J. Chromatogr. Sci..

[44] Ismaiel O., Halquist M.S., El-Mammli M.Y., Shalaby A., Karnes H.T. (2008). Development of a Liquid Chromatography-Negative ESI-Tandem Mass Spectrometry Method for Ibuprofen with Minimization of Matrix Effects Associated with Phospholipids. J. Liq. Chromatogr. Relat. Technol..

[45] Pirro V., Jarmusch A.K., Vincenti M., Cooks R.G. (2015). Direct drug analysis from oral fluid using medical swab touch spray mass spectrometry. Anal. Chim. Acta.

[46] Davies N.M., Anderson K.E. (1997). Clinical Pharmacokinetics of Diclofenac. Clin. Pharmacokinet..

