# Supplementary material for: Spherical Sampler Probes Enhance the Robustness of Ambient Ionization Mass Spectrometry for Rapid Drugs Screening
Source: Molecules. 2022 Jan 30;27(3):945. doi: 10.3390/molecules27030945 (PMC8840626; doi:10.3390/molecules27030945)
Supplement: Supplementary file 1 [file molecules-27-00945-s001.zip › molecules-1548447-supplementary.pdf]

Supplementary Materials

Spherical Sampler Probes Enhance the Robustness of Ambient Ionization Mass Spectrometry for Rapid Drugs Screening

Mariya A. Shamraeva, Denis S. Bormotov, Ekaterina V. Shamarina, Konstantin V. Bocharov, Stanislav I. Pekov, Eugene N. Nikolaev, Igor A. Popov

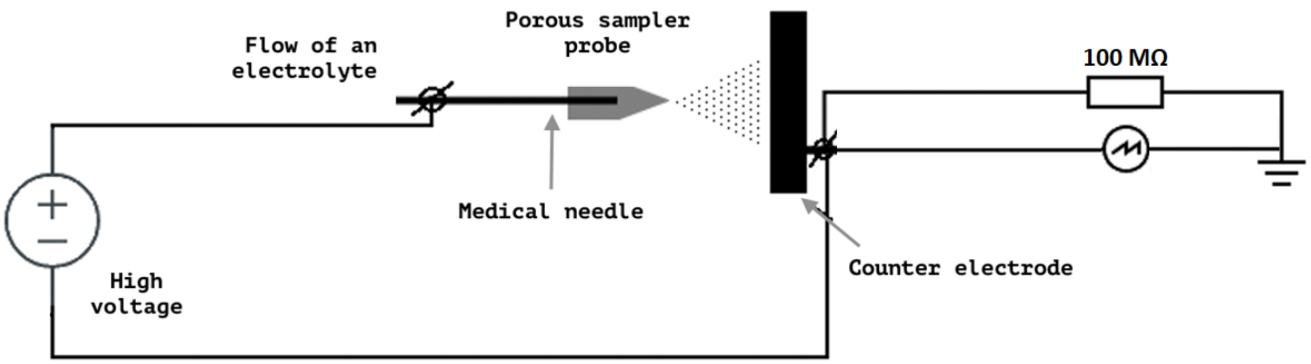

Figure S1. Principal scheme of the setup for CVC determination.

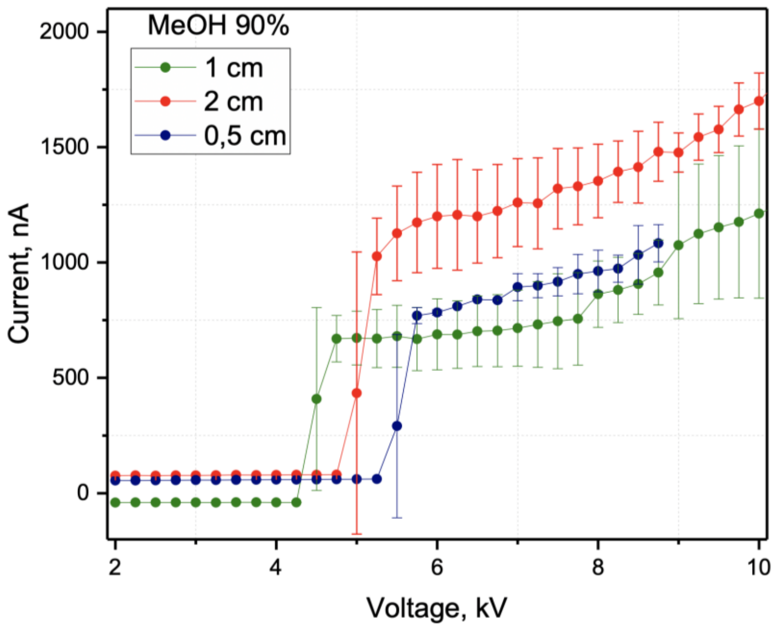

Figure S2. ESI current as a function of an applied voltage at the range 2-10,5 kV for different distances for 9:1 MeOH:H_2O.

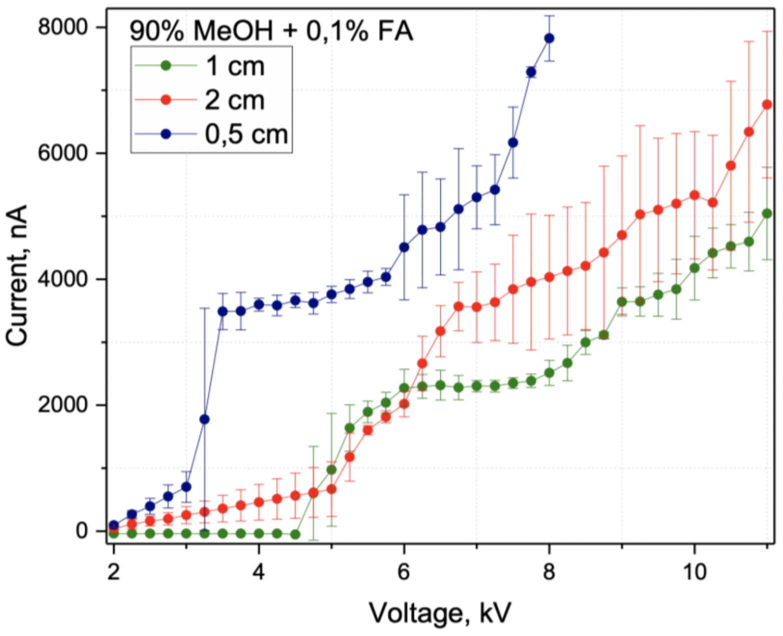

Figure S3. ESI current as a function of an applied voltage at the range 2-10,5 kV for different distances for 9:1 MeOH:H_2O + 0,1% FA.

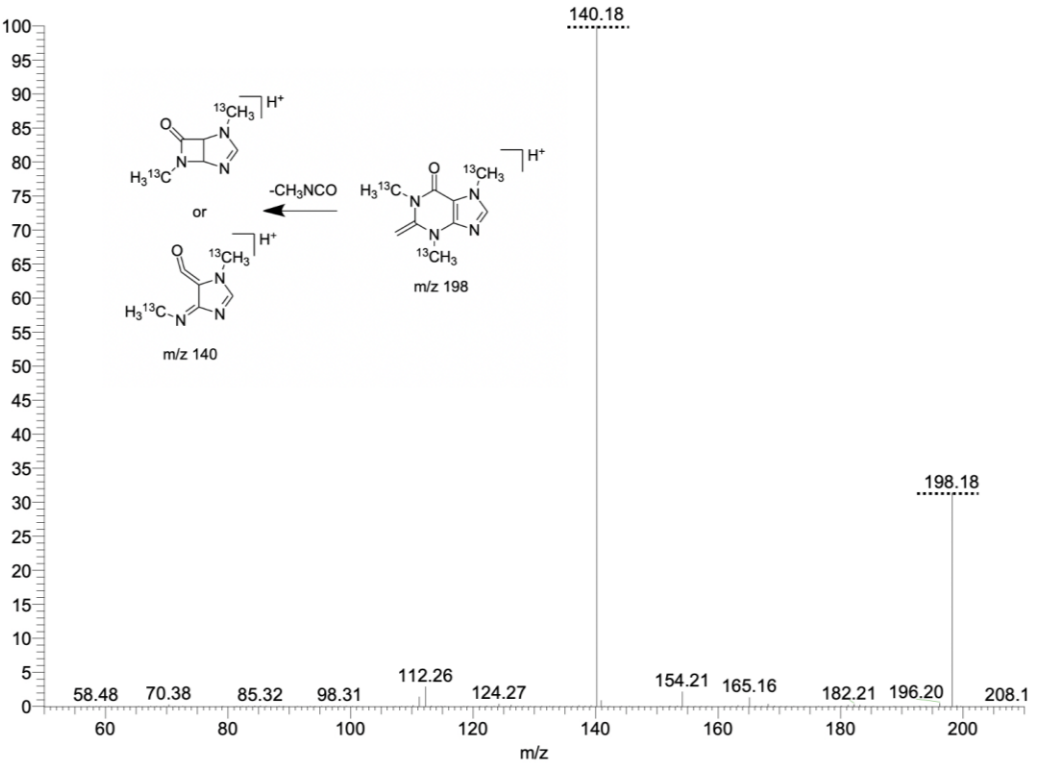

140.18
198.18
-CH3NCO
or
m/z 140
m/z 198
58.48
70.38
85.32
98.31
112.26
124.27
154.21
165.16
182.21
196.20
208.1
m/z

a

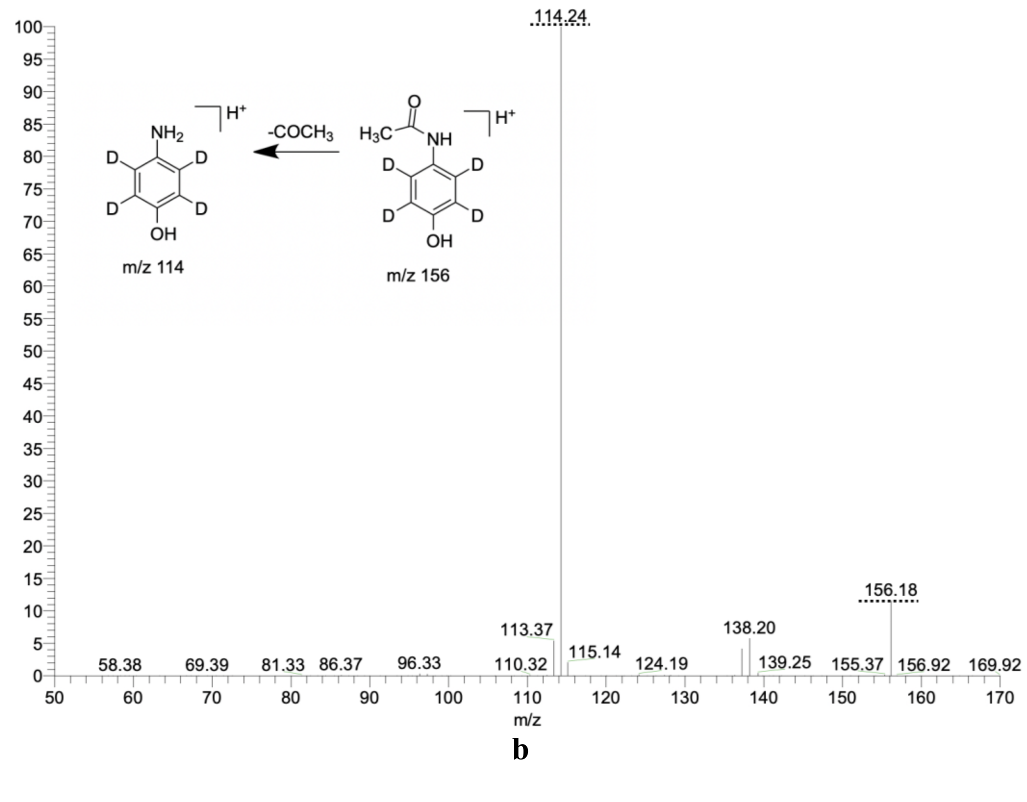

114.24
156.18
-COCH3
m/z 114
m/z 156
58.38
69.39
81.33
86.37
96.33
110.32
113.37
115.14
124.19
138.20
139.25
155.37
156.92
169.92
m/z

b

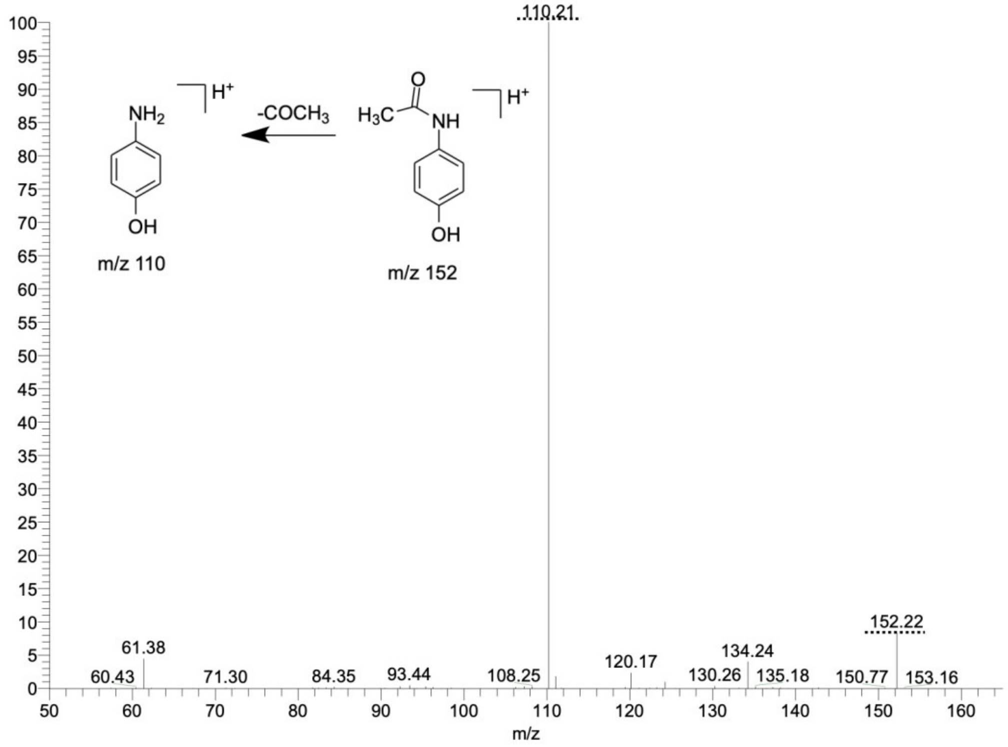
110.21
152.22
134.24
120.17
61.38
60.43
71.30
84.35
93.44
108.25
130.26
135.18
150.77
153.16
-COCH3
m/z 110
m/z 152
m/z

c

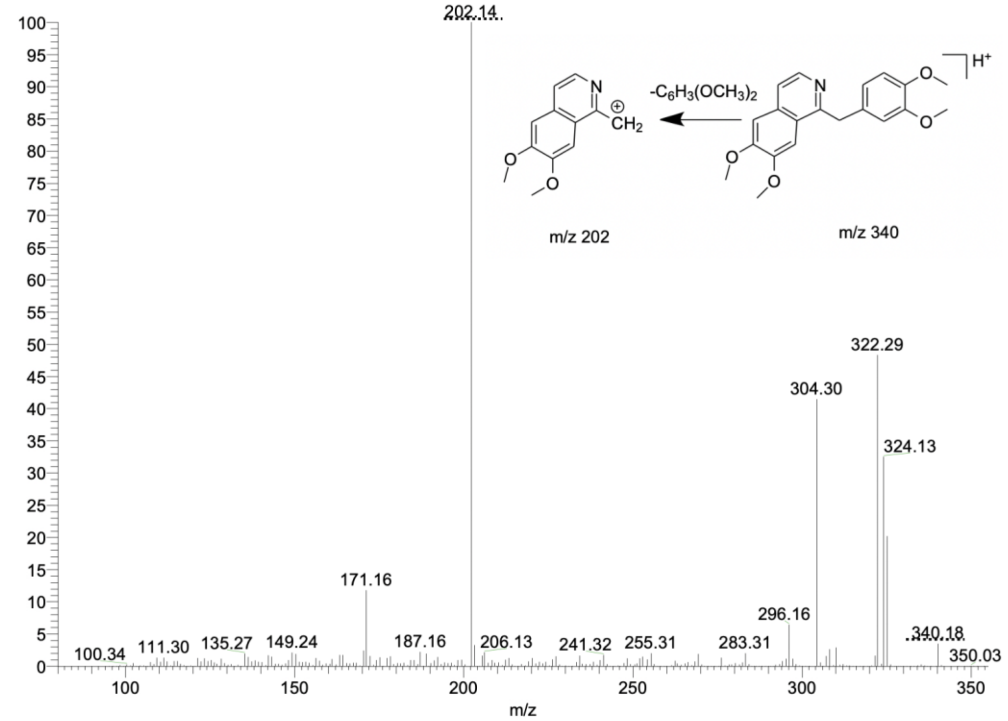
202.14
-C6H3(OCH3)2
m/z 202
m/z 340
322.29
304.30
324.13
171.16
296.16
340.18
350.03
100.34
111.30
135.27
149.24
187.16
206.13
241.32
255.31
283.31
m/z

d

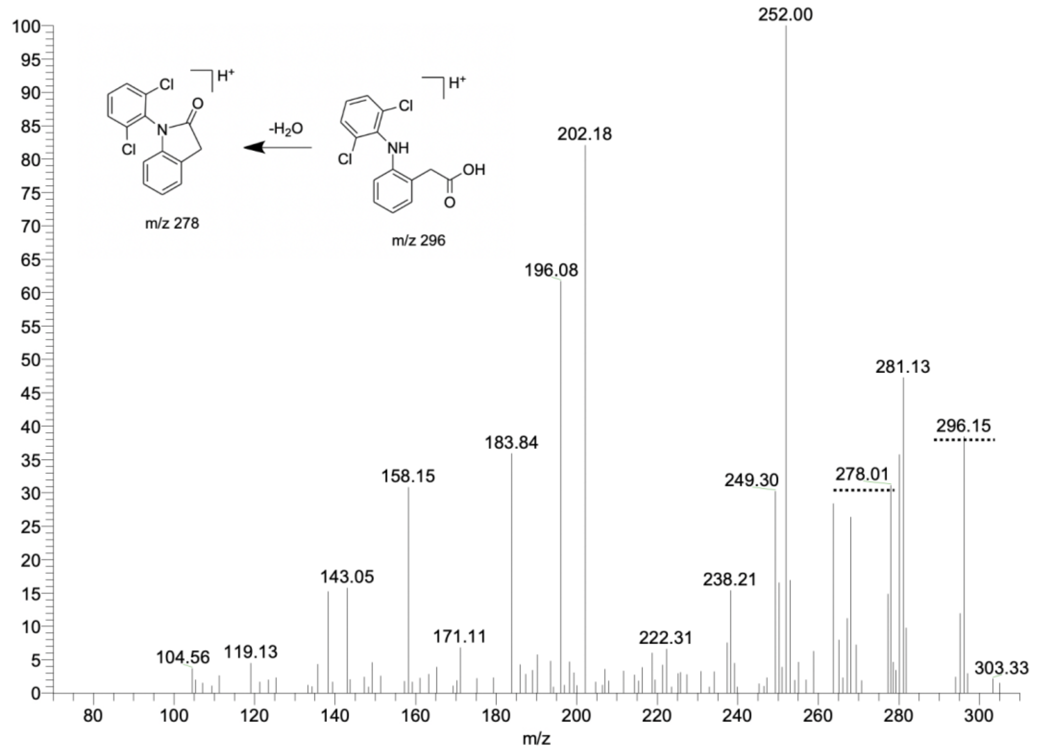

e

Figure S4. Fragmentation ion spectrum of using CID as collected on a LTQ-ion trap in positive mode:
a - caffeine-trimethyl-$^{13}C_3$; **b** - acetaminophen-d_4; **c -** acetaminophen; **d -** papaverine; **e** - diclofenac

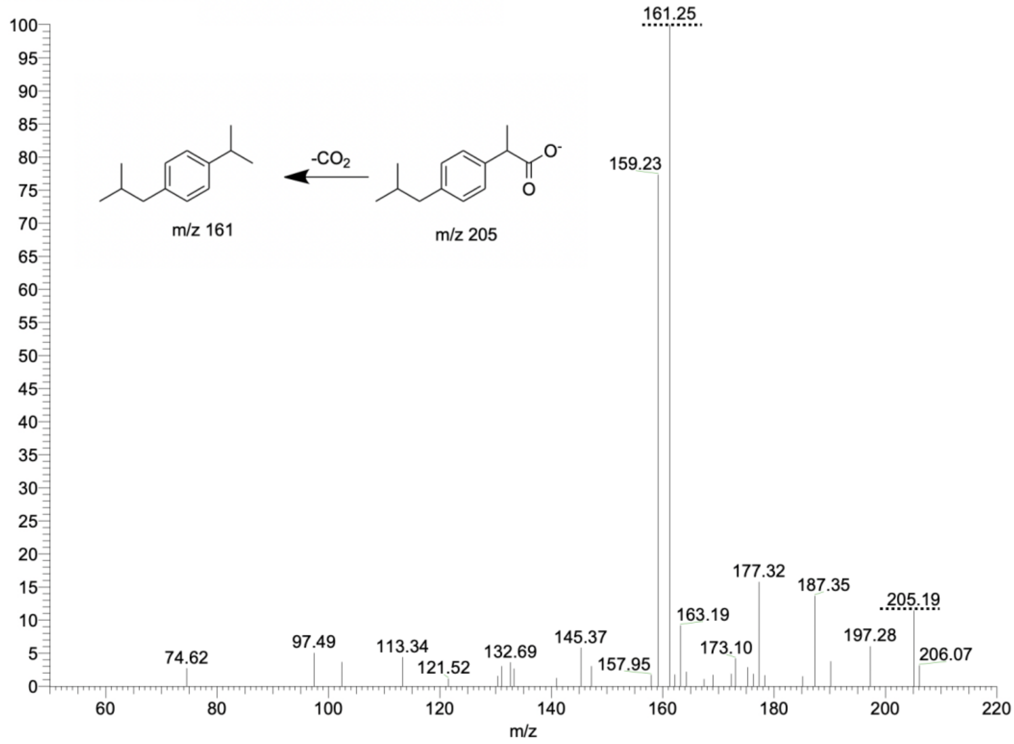

161.25
159.23
-CO2
m/z 161
m/z 205
177.32
187.35
205.19
163.19
173.10
197.28
206.07
74.62
97.49
113.34
121.52
132.69
145.37
157.95
m/z

a

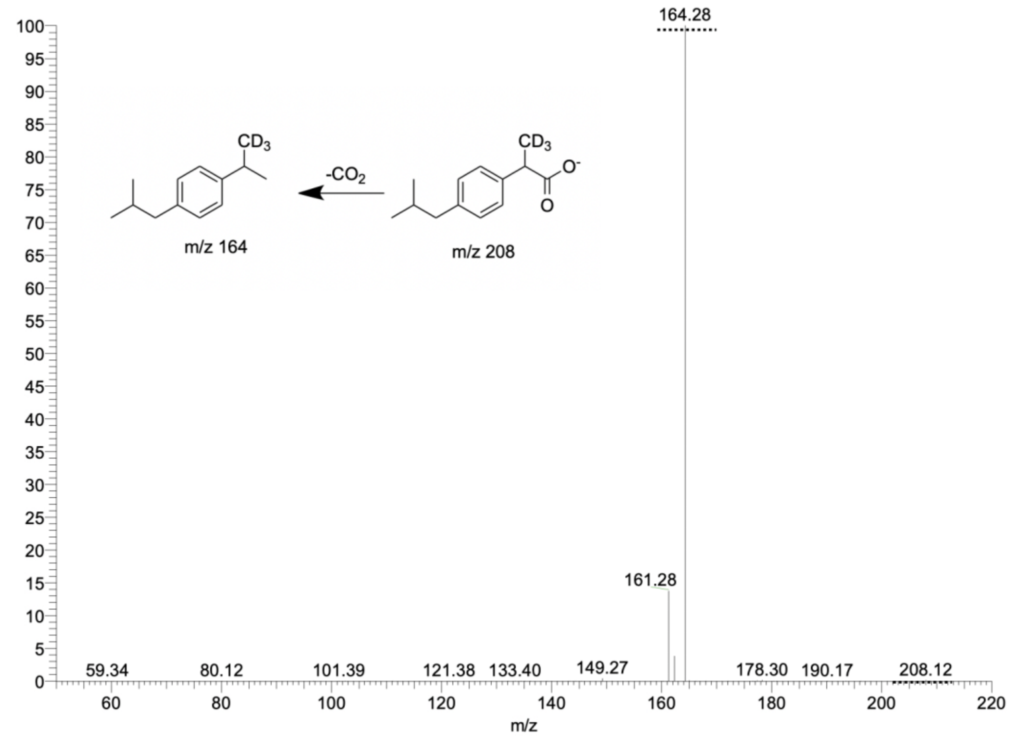

164.28
CD3
-CO2
m/z 164
m/z 208
161.28
59.34
80.12
101.39
121.38
133.40
149.27
178.30
190.17
208.12
m/z

b

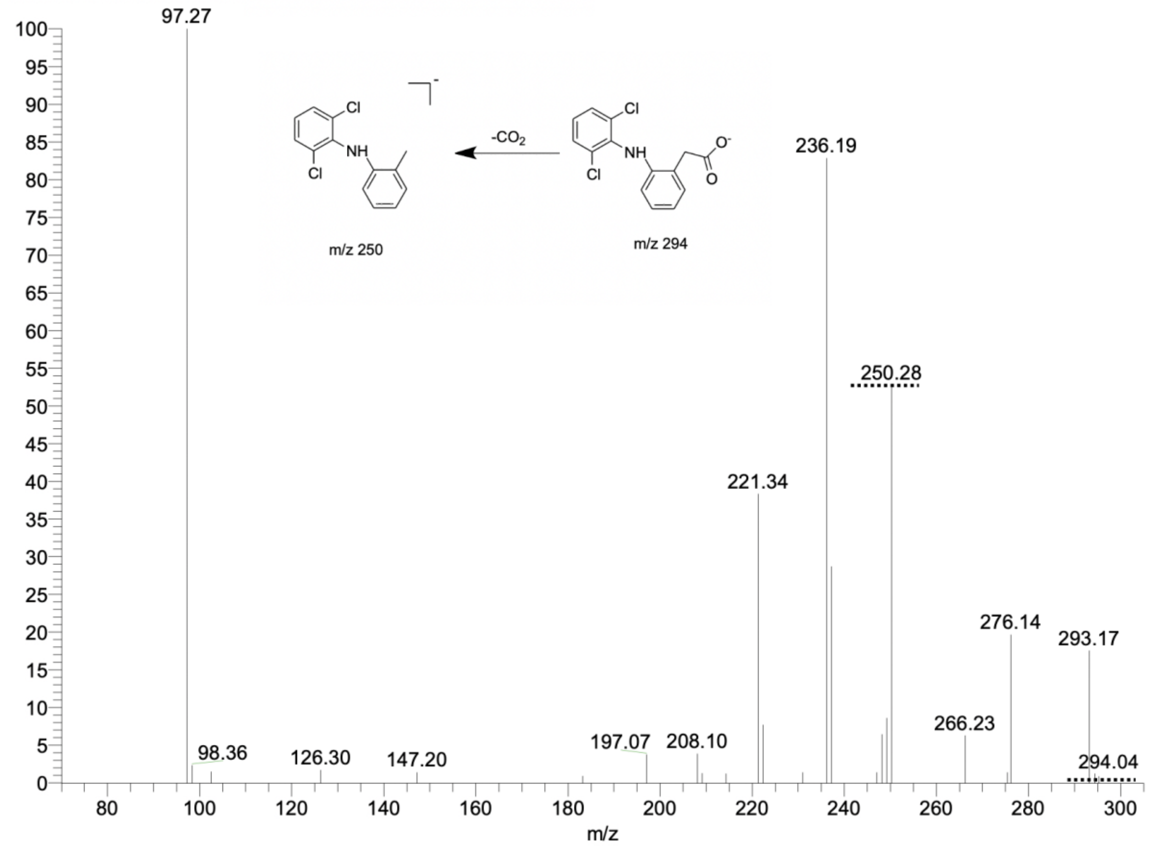

c

Figure S5. Fragmentation ion spectrum of using CID as collected on a LTQ-ion trap in negative mode:
a - ibuprofen; **b** - ibuprofen-d$_3$; **c -** diclofenac;

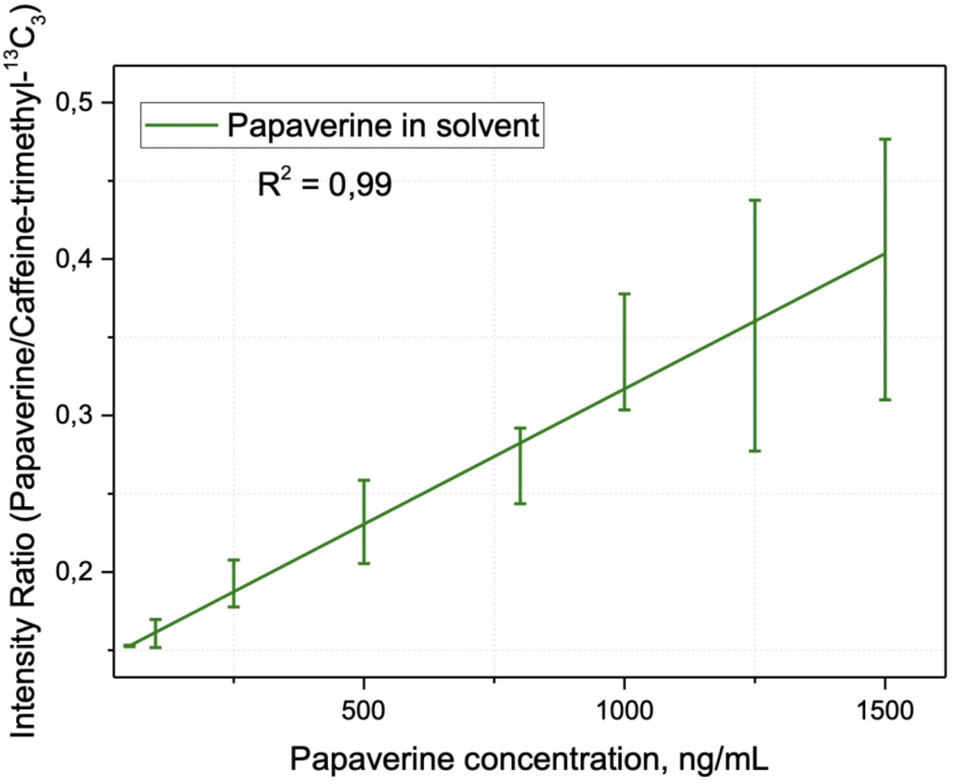

a

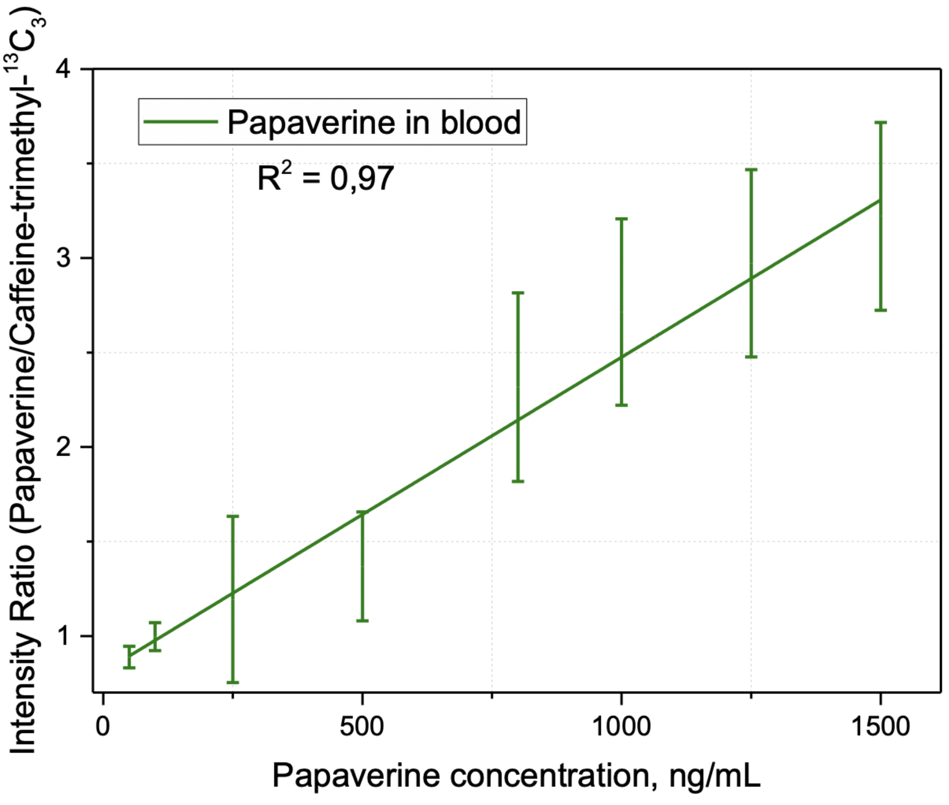

b

Figure S6. Calibration curves in positive mode (p-value < 0,05): **a** Papaverine with Caffeine-trimethyl-$^{13}C_3$ as an internal standard. Matrix: 9:1 (v:v) MeOH:H_2O; R^2 = 0, 99. **b** Papaverine with Caffeine-trimethyl-$^{13}C_3$ as an internal standard. Matrix: dried blood; R^2 = 0, 97.

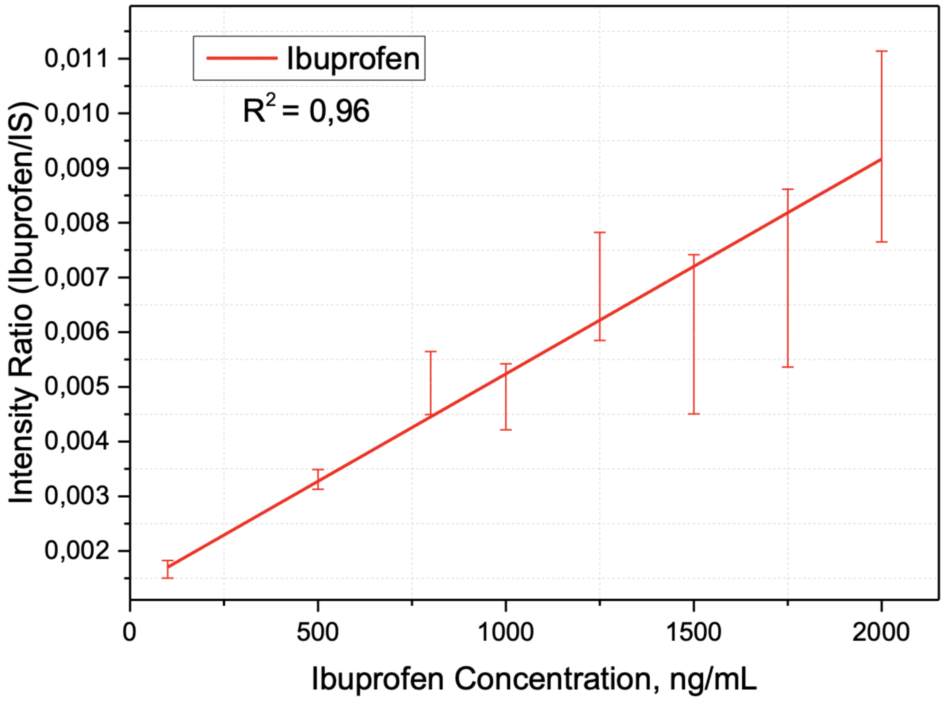

a

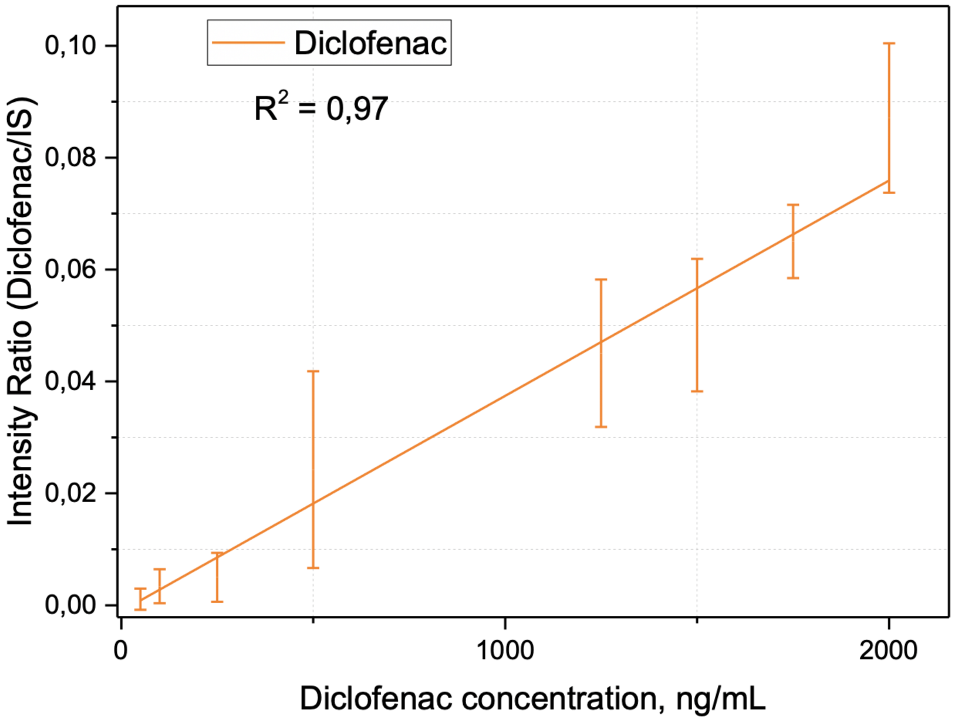

b

Figure S7. Calibration curves in negative mode (p-value < 0,05):
a Ibuprofen with Ibuprofen-d_3 as an internal standard.
Matrix: 1:1 (v:v) MeOH:i-PrOH; R^2 = 0, 96.
b Diclofenac with Ibuprofen-d_3 as an internal standard.
Matrix: 1:1 (v:v) MeOH:i-PrOH,; R^2 = 0, 97.

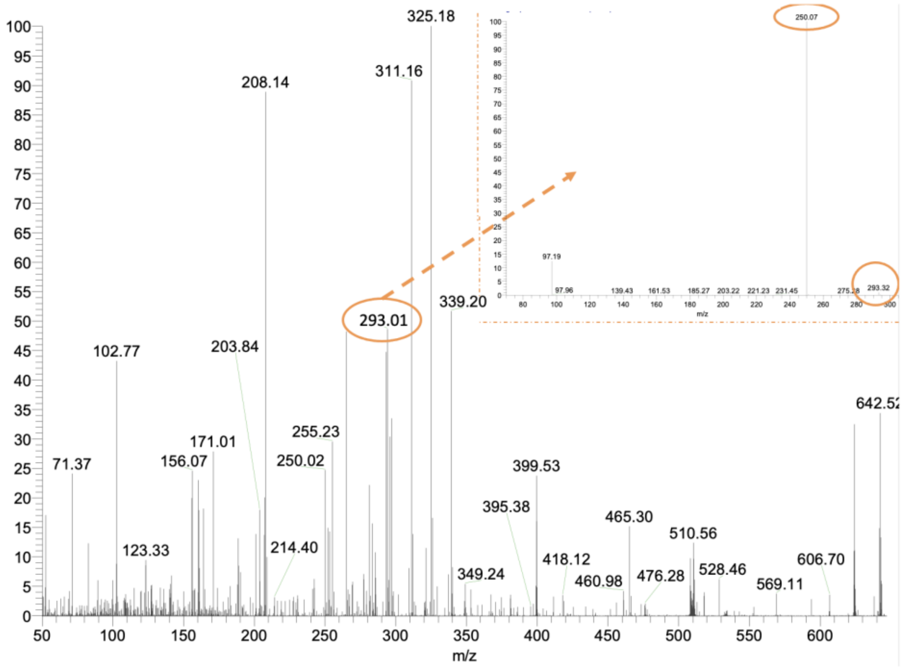

a

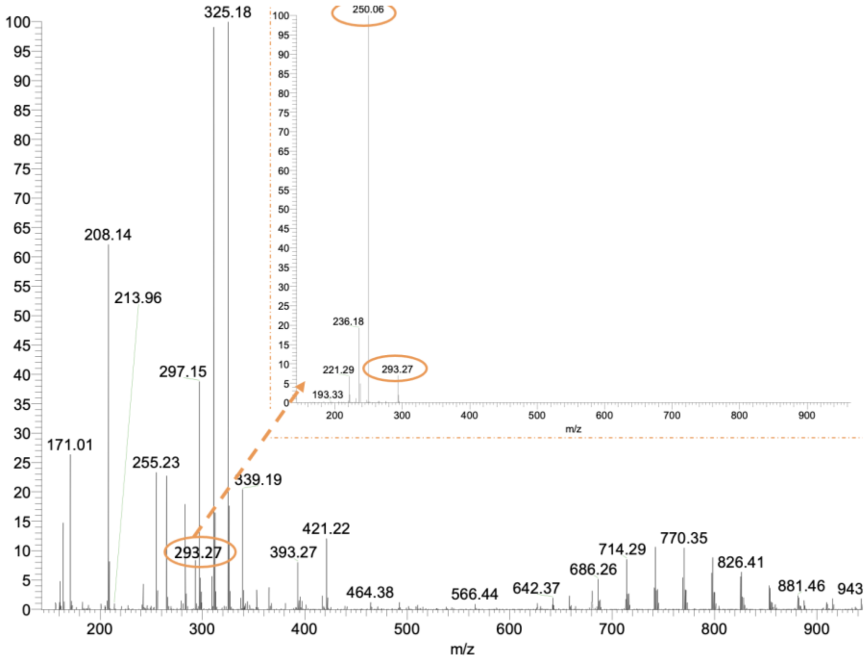

b

Figure S8. Mass spectra of diclofenac in sebum after 15 minutes after skin anointing (**a**); and spiked in whole blood (**b**). Negative ion mode;

Table S1. Analyzed chemical structures and monitored product ions.

Therapeutic drug	Precursor ion, m/z	Monitored fragment ion, m/z
Acetaminophen	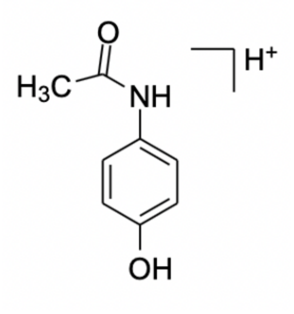 152	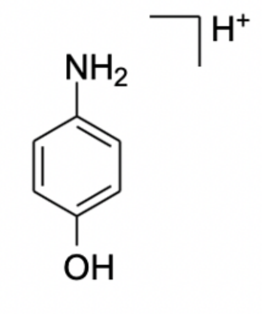 110
Papaverine	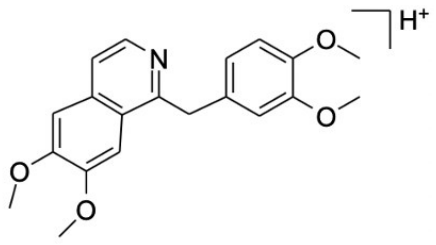 340	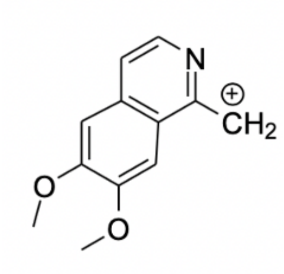 202
Diclofenac	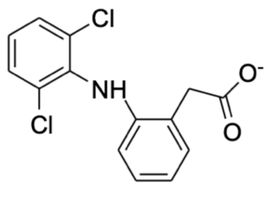 294	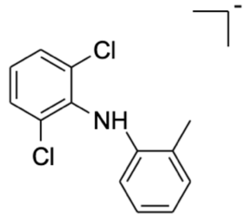 250
Ibuprofen	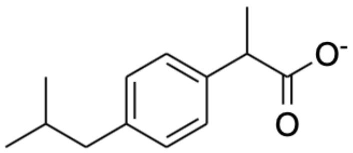 205	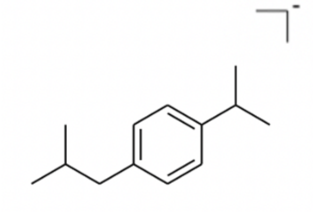 161
**Internal standards**		
Acetaminophen-d_4	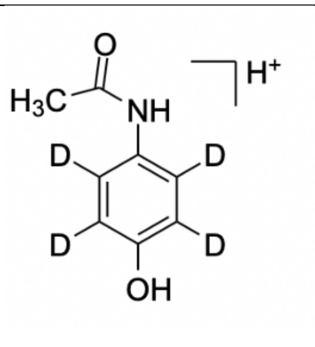 156	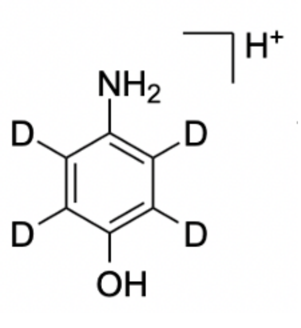 114

Caffeine-trimethyl-$^{13}C_3$	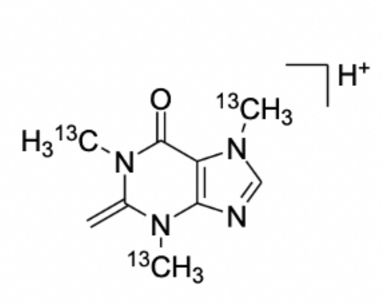 198	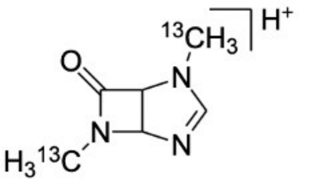 or 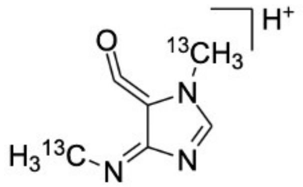 140
Ibuprofen-d_3	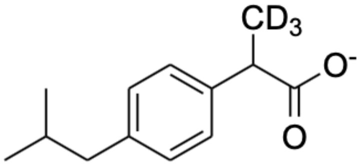 208	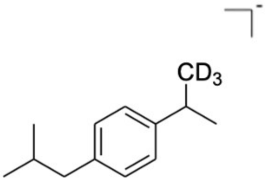 164
